# Weibel-Palade body size modulates the adhesive activity of its von Willebrand Factor cargo in cultured endothelial cells

**DOI:** 10.1038/srep32473

**Published:** 2016-08-31

**Authors:** Francesco Ferraro, Silva Mafalda Lopes da, William Grimes, Hwee Kuan Lee, Robin Ketteler, Janos Kriston-Vizi, Daniel F. Cutler

**Affiliations:** 1 MRC Laboratory for Molecular Cell Biology, UCL, Gower Street, London, WC1E 6BT, United Kingdom; 2 Imaging Informatics Division, Bioinformatics Institute, A*STAR 30 Biopolis Street #07-01, Matrix, Singapore 138671

## Abstract

Changes in the size of cellular organelles are often linked to modifications in their function. Endothelial cells store von Willebrand Factor (vWF), a glycoprotein essential to haemostasis in Weibel-Palade bodies (WPBs), cigar-shaped secretory granules that are generated in a wide range of sizes. We recently showed that forcing changes in the size of WPBs modifies the activity of this cargo. We now find that endothelial cells treated with statins produce shorter WPBs and that the vWF they release at exocytosis displays a reduced capability to recruit platelets to the endothelial cell surface. Investigating other functional consequences of size changes of WPBs, we also report that the endothelial surface-associated vWF formed at exocytosis recruits soluble plasma vWF and that this process is reduced by treatments that shorten WPBs, statins included. These results indicate that the post-exocytic adhesive activity of vWF towards platelets and plasma vWF at the endothelial surface reflects the size of their storage organelle. Our findings therefore show that changes in WPB size, by influencing the adhesive activity of its vWF cargo, may represent a novel mode of regulation of platelet aggregation at the vascular wall.

The relationship between an organelle’s size and its function has become recognized as an important element in the modulation of cellular behaviour, both in physiology and pathology^1^,^2^,^3^. We have been investigating this phenomenon in endothelial cells, using their pro-haemostatic granules as a model system of clinical relevance. The major cargo of these granules, the Weibel-Palade bodies (WPBs), is the haemostatic protein von Willebrand Factor (vWF)^4^. Formed by concatamers (often referred to as multimers) of its ~270 kDa monomer, plasma vWF is the largest soluble protein in vertebrates and plays a fundamental role in primary haemostasis^5^. This function depends on the multimeric structure of vWF, which in the circulation is stretched by shear to expose binding sites that anchor it at the vascular wall and recruit platelets, thus localizing formation of primary haemostatic plugs^6^,^7^. The fundamental role of vWF in haemostasis is highlighted by genetic or acquired deficiencies, qualitative or quantitative, that cause von Willebrand disease (vWD), the most common inherited bleeding disorder in humans^5^. vWF’s contribution to pro-thrombotic conditions is dramatically demonstrated by thrombotic thrombocytopenic purpura (TTP), a condition in which the ultra-large vWF multimers released by endothelial cells are not reduced in size by the circulating metalloprotease ADAMTS13. Genetic deficiency or autoimmune inhibition of this protease results in the formation of disseminated thrombi in the microcirculation, a life-threatening condition.

Within WPBs, highly multimerized vWF is coiled into tubules^8^; a structural arrangement that imparts the elongated, cigar-like morphology of these secretory granules and is necessary to the orderly release of vWF^9^. This arrangement requires that the structures of vWF and of the WPB are closely related, a major reason why the relationship of organelle size and function are especially transparent in this system. In addition, in cultured endothelial cells, WPBs are present with lengths between 0.5 and 5 μm, providing a large range for study.

vWF plays a multifaceted role in platelet adhesion and thrombus formation. vWF is synthesized not only by endothelial cells but also megakaryocytes and stored in platelets; however, experimental evidence shows that the endothelial vWF contribution to haemostasis outweighs that of platelets^10^,^11^ Soluble plasma vWF, derived from ADAMTS13 processing of the highly multimerized vWF secreted from endothelial cells can bind to substratum-adhered vWF, a homotypic association regulated by shear stress^12^,^13^. At pathologically high shear rates, soluble vWF can self-associate on collagen coated substrata (a model of the sub-endothelial matrix) and generates, in a concentration-dependent manner, string-like structures capable of recruiting platelets^14^. Within physiological shear stress values observed in the venous vasculature (2.5–20 dynes/cm^2^ or 0.25–2 MPa)^15^, vWF released by exocytosis of WPBs forms transient strings that are tens to hundreds of microns long^16^. These vWF strings result from the self-association of ultra-large (UL) vWF multimers into cables that are anchored at the endothelial surface and have been visualized at the ultra-structural level^17^. In order to form strings, UL-vWF multimers likely interact side by side and in an out-of-register fashion^18^, the only possible arrangement that explains both the cable structure and the vast length these structures can achieve. vWF strings, by adhering to the cell surface, provide a transient scaffold for platelet recruitment before being degraded by ADAMTS13^16^,^19^. Platelet-decorated vWF strings can further increase in number and complexity in the presence of soluble plasma vWF^18^,^20^. vWF function is thus closely linked to its quaternary, supra-multimeric structure.

We recently investigated how WPBs acquire their size^21^. During their biogenesis, the size of WPBs is dictated by the general architecture of the Golgi apparatus. A continuous Golgi ribbon allows for co-packaging of vWF cargo units, called “quanta”, into nascent WPBs. The final size of a WPB, therefore, depends on the number of quanta that are encased into each organelle at biogenesis. Unlinking (a.k.a. fragmentation) of the Golgi ribbon prevents such co-packaging, resulting in formation almost exclusively of short WPBs, termed mini-WPBs (Fig. 1a)^21^. In the same study, the functional significance of organelle size was investigated. Long WPBs (which we define as >2 μm) represent a minority of the organelle population but contain a significant fraction of the vWF stored by endothelial cells (Fig. 1b). This observation raised the possibility that long WPBs might be disproportionally important in defining the activity of the vWF released by endothelial cells. Indeed, we found that at exocytosis mini-WPBs generate shorter platelet-decorated vWF strings on the endothelial surface. These findings raised the possibility that endothelial cells may respond to physiological and pathological cues by modulating their haemostatic phenotype through changes in the size of these secretory organelles^21^. On one side, endothelial dysfunction might result in the production of a higher fraction of long WPBs and thus lead to increased pro-thrombotic propensity; on the other, a higher proportion of short organelles could result in an antithrombotic phenotype. At present, little is known about whether and how WPB size is regulated and our conclusions were based on experimental manipulations to generate mini-WPBs that are not physiological. Here, to validate our hypothesis, we addressed whether WPB size can be pharmacologically regulated. We find that statin treatment of cultured endothelial cells reduces the size of their WPBs, and like other treatments with the same effect, this impacts on both platelet recruitment and the recruitment of circulating plasma vWF to the endothelial surface. These new results lend further support to the idea that WPB size can play a role in the regulation of platelet aggregation.

## Results

### Statin treatment of endothelial cells reduces the size of WPBs and the length of platelet-decorated vWF strings

**Figure 1:**
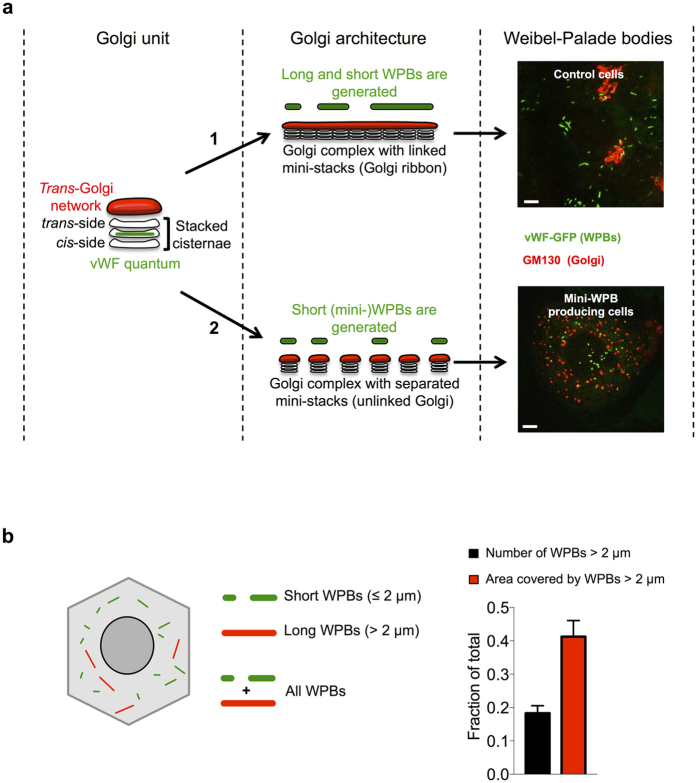
How WPB size is determined and its relevance. (**a**) The functional unit of the Golgi apparatus is the mini-stack, a pile of flat membrane cisternae. At its entry side (the *cis*-side) the mini-stack receives secretory cargo from the endoplasmic reticulum and at its exit side (*trans*-side) this cargo is delivered to the *trans*-Golgi network. At the *trans*-Golgi network, cargos are packaged into transport carriers and sorted to their final destination within the cell. We have found that WPB size is determined by the structural status of the Golgi apparatus^21^. Mini-stacks, whose diameter ranges between 500 and 1000 nm, impart a limit to the size of the transiting vWF cargo, generating what we have defined vWF “quanta”. When the mini-stack are linked to form the structure known as Golgi ribbon (route 1), both short and long WPBs are formed, since vWF quanta can be co-packaged together at the *trans*-Golgi network. When the mini-stacks are unlinked (route 2), co-packaging of quanta is prevented and short WPBs are produced. The micrographs exemplify these conditions. HUVECs were transfected with vWF-GFP to label the WPBs being made during the treatment. In untreated cells (control cells) mini-stacks are linked into a ribbon (GM130) and WPBs of various sizes are generated. In cells with unlinked mini-stacks (route 2, obtained by treatment with nocodazole), only short (or mini-) WPBs are produced. Scale: 5 um. (**b**) WPBs have a typical cylindrical morphology and in untreated cultured HUVECs (as the Control in panel a) their length ranges between 0.5 and 5 μm^21^. The total area covered by WPBs indirectly measures the vWF they store. Morphological analysis indicates that long WPBs (defined as those longer than 2 μm; red-coded in the cartoon) represent a minor fraction of total number of organelles but account for a large fraction of the area they cover (bar graph, black vs red bar). Therefore, while a minority, long WPBs store a significant fraction of vWF. The quantifications displayed were obtained by High-throughput morphometric analysis (see text) of 4 sets (means ± SD) of untreated cells. Number of WPBs measured ~10^5^.

**Figure 2:**
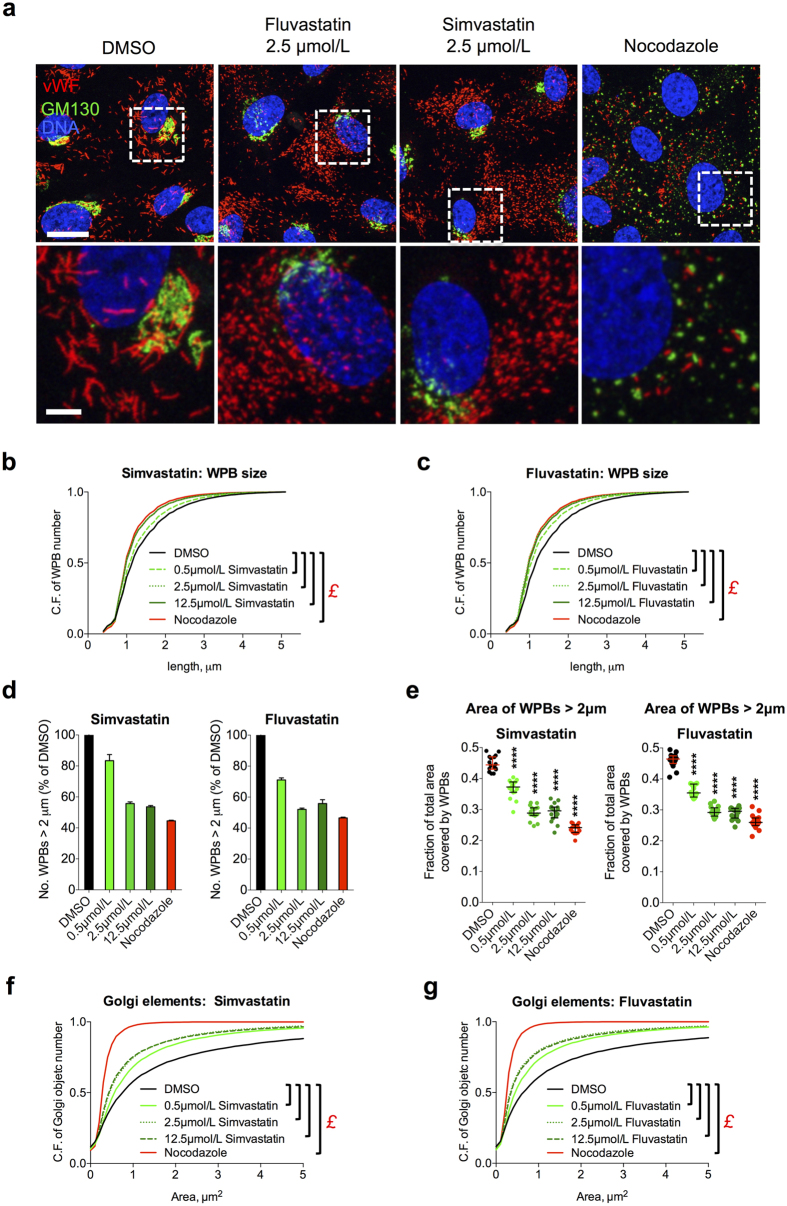
Statins induce production of mini-WPBs in HUVECs. (**a**) HUVECs were grown for 24 h in medium containing vehicle (DMSO), statins or nocodazole at the indicated concentrations before being fixed and processed for immunofluorescence of the indicated markers (vWF for WPBs, GM130 for Golgi complexes). Nocodazole was used as a positive control treatment to induce mini-WPBs. Scale bar: 20 μm; magnified insets: 5 μm. (**b**,**c**) High-throughput morphometric (HTM) analysis of the whole population of WPBs in HUVECs treated for 24 h as indicated. Cumulative frequency (C.F.) plots of the total number of WPBs as a function of the organelles’ length. Relative to the control plot (DMSO), left-shifted curves indicate WPB size shortening, as shown by the nocodazole traces (the positive control for mini-WPB production). The number of WPBs analysed was ~10^5^ per condition. £, *P* < 10^−15^ for the indicated pairwise comparisons (Mann-Whitney test). (**d**) The fraction of long WPBs (>2 μm) relative to DMSO controls is shown. Bars: mean ± range for two replicate HTM experiments with simvastatin and fluvastatin. (**e**) HTM analysis of the area covered by long organelles (>2 μm) as a fraction of the total area covered by all WPBs (see Fig. 1b) in HUVECs treated as indicated for 24 h. N = 16 for each treatment; median and interquartile ranges are shown; ****P < 0.0001. Comparisons are to the DMSO control (Mann-Whitney test). (**f**,**g**) HTM analysis of the whole population of the Golgi objects in cells treated with the indicated concentrations of simvastatin and fluvastatin. Cumulative frequency (C.F.) plots of the number of Golgi objects as a function of the their area. The number of Golgi objects analysed was ~10^4^−10^5^ in all conditions. £, *P* < 10^−15^ for the indicated pairwise comparisons (Mann-Whitney test).

Statins reduce cholesterol plasma levels and decrease morbidity and mortality in patients with or at risk of cardiovascular disease^22^,^23^,^24^. More recently, *in vitro*, animal and clinical studies have provided evidence that statins have also antithrombotic effects^25^,^26^,^27^,^28^,^29^,^30^,^31^,^32^,^33^,^34^. Given our previous findings about the size of WPBs affecting platelet recruitment at the endothelial surface, and the antithrombotic phenotypes of statins, we investigated the effect on WPB size of this class of drugs. We treated human umbilical vein endothelial cells (HUVECs) for 24 h with two statins, simvastatin and fluvastatin, at concentrations usually used in *in vitro* studies^31^,^35^,^36^,^37^,^38^. Microscopic inspection showed that, at all concentrations tested, both statins reduced the size of WPBs. (Figs 2a and S1a). These effects were measured with an approach that we recently developed and named high-throughput morphometry (HTM), which allows the unbiased morphological quantification of tens to hundreds of thousands of organelles^21^. Induction of mini-WPB production was concentration-dependent: at 2.5 and 12.5 μmol/L both statins shortened the size of WPB to lengths similar to those generated by nocodazole, our positive control for mini-WPB generation, with an intermediate effect at 0.5 μmol/L (Fig. 2b–e). These effects on WPB size are explained by the unlinking of the Golgi ribbon in statin treated cells (Fig. 2a the Golgi marker GM130, S1a and 2f,g; also refer to Fig. 1a). Statin treatment generated Golgi fragments that clustered in proximity to the nucleus, similar to the treatment with acidic pH/acetate (Figs 2a, S1a and 5g), another treatment that we have previously shown to reduce WPB size through Golgi ribbon unlinking^21^. The Golgi unlinking induced by statins was morphologically different from that caused by nocodazole; statins do not induce microtubule depolymerisation, the basis for Golgi mini-stack scattering seen with nocodazole (Figs 2a and S1a,b). We conclude that statins cause WPB size reduction through unlinking of the Golgi ribbon, in agreement with the current model for the biogenesis of these organelles (Fig. 1a)^21^.

**Figure 3:**
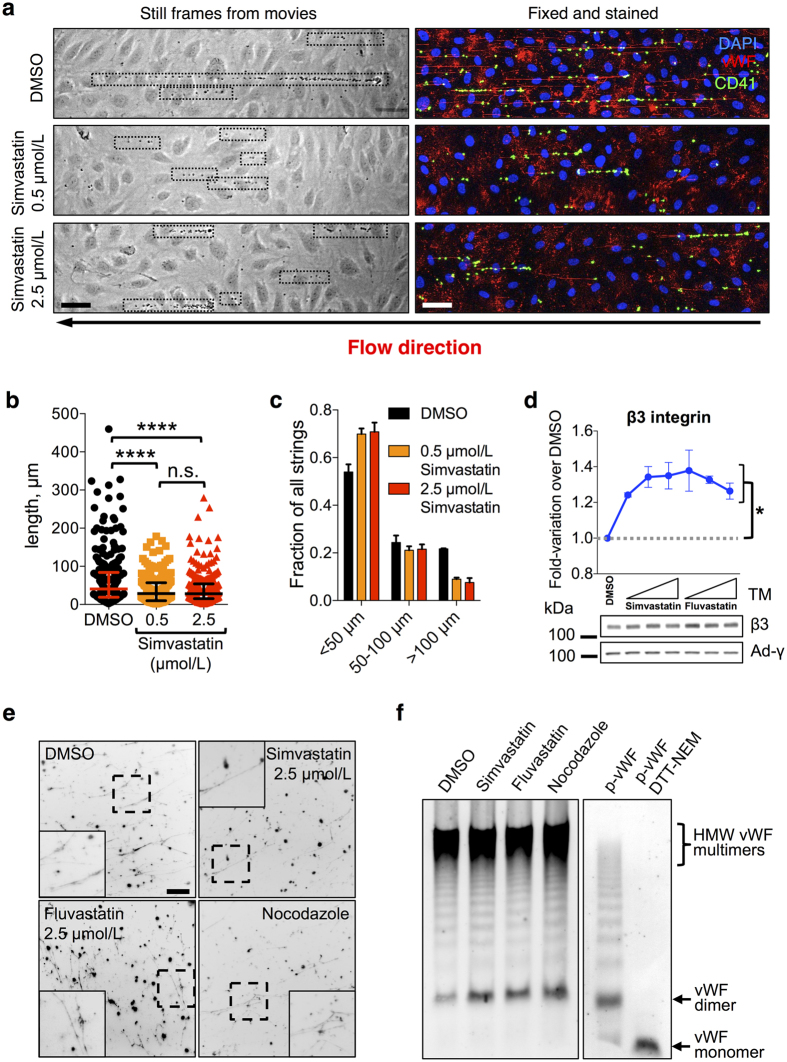
Statin treatment reduces platelet recruitment to the endothelial surface. (**a**) HUVEC monolayers treated with vehicle (DMSO), 0.5 or 2.5 μmol/L simvastatin for 24 h were perfused with washed platelets from healthy non-medicated volunteers in the presence of histamine (100 μmol/L) to stimulate vWF secretion. Left, video-microscopy frames taken during perfusion show platelet-decorated strings (highlighted by dotted boxes). Right, samples were fixed and immuno-stained to visualize vWF strings and platelets (CD41). Scale bar: 50 μm. (**b**) Analysis of the length of platelet-decorated vWF strings, pooled from two independent experiments, in duplicate for each treatment. Median and interquartile ranges are shown. Number of strings measured: DMSO, 271; simvastatin 0.5 μmol/L, 217; simvastatin 2.5 μmol/L, 278. ****P < 0.0001 (Mann-Whitney test). (**c**) Statin treatment primarily affected the longest (>100 μm) strings. Error bars represent the range of variation for the two experiments. (**d**) Integrin β3, which in endothelial cell is present in complex with integrin αν and functions as an anchor for vWF strings, was quantified from western blots by densitometry following incubation for 24 h with 0.5, 2.5 and 12.5 μmol/L statins (indicated by the triangle symbols). n = 3; mean ± SEM. *P < 0.05 or lower (not shown for simplicity) for all treatments compared to DMSO control (Student’s t-test). γ-Adaptin (Ad-γ) was used as a loading control. (**e**) Cell-free WPBs prepared from cells treated for 24 h as indicated were detergent-permeabilised and the resulting vWF filaments immuno-stained and imaged (insets show magnifications of the dashed boxes). Scale bar: 25 μm. (**f**) vWF multimerization in cell-free WPBs was analysed by agarose gel electrophoresis. Highly multimerized vWF is indicated (high-molecular weight, HMW). The multimeric patterns of plasma vWF (p-vWF) untreated or reduced (dithiothreitol, DTT) and alkylated (N-ethylmaleimide, NEM) to generate monomers are shown for comparison.

If our hypothesis that WPB size correlates with the length of platelet-recruiting adhesive strings that vWF forms under flow on the endothelial surface is correct, then statin incubation should result in a reduction in the size of these structures. Statins have antiplatelet effects *in vitro, ex vivo,* in animal models and in patients^29^,^32^,^39^. Therefore, in order to distinguish their effects on endothelial cells from those on platelets, we used washed platelets from healthy non-medicated volunteers for our experiments. HUVECs treated with simvastatin at 0.5 and 2.5 μmol/L (concentrations that, respectively, induce submaximal and maximal WPB shortening) and challenged with histamine generated shorter platelet-decorated vWF strings (Fig. 3a,b), with the longest strings (>100 μm) being especially affected (Fig. 3c).

This reduced length of the strings might be due to a reduced capability of secreted vWF to adhere to the endothelial surface. A known anchor of vWF strings to the endothelial surface is αvβ3 integrin^17^. Statin treatment might down-regulate of αvβ3 integrin, thus reducing the ability of the endothelial surface to bind secreted vWF. In fact, αvβ3 levels were slightly up-regulated following treatment with both simvastatin and fluvastatin (Fig. 3d), ruling out that the reduced platelet recruitment was due to a defect in string anchoring to the endothelial surface.

Shorter vWF strings could also result from a reduction in vWF multimerization^40^. The possibility thus exists that the production of mini-WPBs might influence the multimeric state of the vWF they store and therefore the length of the resulting strings. To address this, we prepared cell-free WPBs, from control-, statin- and nocodazole-treated cells. Permeabilization of cell-free WPBs results in the formation of vWF filaments, the likely precursors of the strings formed upon exocytosis^21^. During WPB biogenesis, vWF undergoes pH- and Ca^2+^-dependent conformational rearrangements necessary for inter-dimer disulphide formation and therefore required for multimerization^41^,^42^. Cell-free organelles from control and mini-WPB producing cells were equally capable of releasing vWF filaments *in vitro* (Fig. 3e), indicating that vWF folding and inter-dimer disulphide bond formation were unaffected by organelle size. In addition, WPB size did not have any obvious effect on the multimeric pattern of vWF, with highly multimerized protein present in comparable abundance in all conditions (Fig. 3f). These data rule out that the mini-WPBs generated following statin or nocodazole treatment impair vWF multimerization. Therefore, changes in vWF multimer size within mini-WPBs cannot explain the reduced length of the platelet-decorated strings. In summary, pharmacological treatment can prompt changes in WPB size and thus influence the adhesive properties of its haemostatic cargo, vWF, towards platelets.

### The role of KLF2 in statin-mediated effects on WPB size

**Figure 4:**
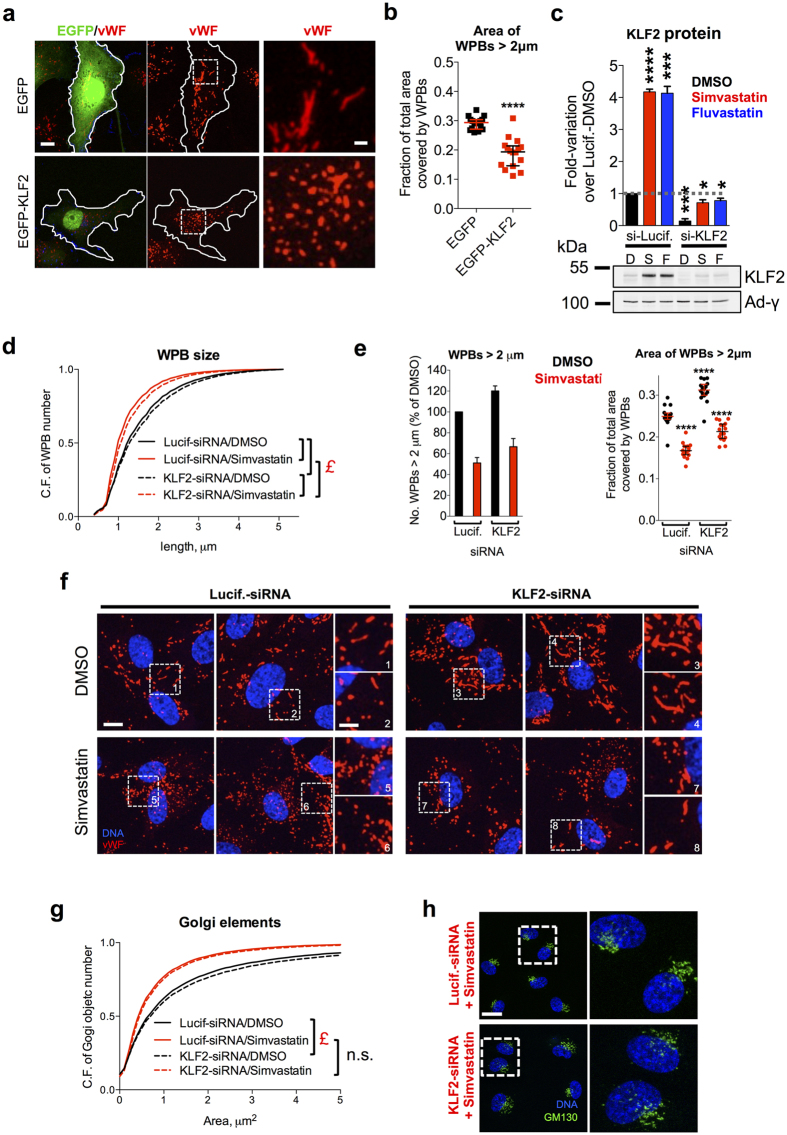
KLF2 and statin mediated effects on WPB size. (**a**) EGFP and EGFP-KLF2 overexpressing HUVECs. Scale bar, 10 μm; inset magnifications: 2 μm. (**b**) HTM analysis showing the fraction of the WPB area covered by long organelles in the subpopulations of EGFP or EGFP-KLF2 overexpressing HUVECs; ****P < 0.0001 (Mann-Whitney test). (**c**) HUVECs were treated with Luciferase or KLF2 targeting siRNAs and after 24 were incubated with DMSO (**D**), 2.5 μmol/L simvastatin (S) or 2.5 μmol/L fluvastatin (**F**) for 24 h before cell lysis. Equal protein amounts were analysed as described for Fig. 3d. Bars represent means ± SEM from 3 experiments. *P < 0.05; ***P < 0.001; ****P < 0.0001 (Student’s test). (**d**) HTM analysis of WPB size in Luciferase- and KLF2-siRNA treated HUVECs incubated with vehicle (DMSO) or 2.5 μmol/L simvastatin for 24 h. Cumulative frequency of WPB number as a function of size (note the right-shift of KLF2 curves in both conditions). The number of WPBs analysed was ~10^5^ per condition. £, *P* < 10^−15^ for the indicated pairwise comparisons (Mann-Whitney test). (**e**) Left, the fraction of long WPBs (>2 μm) was expressed as % of the Luciferase-siRNA/DMSO controls. Bars, mean ± range for two replicate experiments. Right, the fraction of the WPB area covered by long organelles; ****P < 0.0001 (Mann-Whitney test); comparisons are with the Luciferase-siRNA/DMSO controls. (**f**) WPBs in Luciferase- and KLF2-siRNA treated cells after 24 h treatment with DMSO or 2.5 μmol/L simvastatin. Scale bar: 10 μm; inset magnifications: 2 μm. (**g**) HTM analysis of Golgi objects. The numbers of Golgi object analysed were similar to those in Fig. 2e,f. £, *P* < 10^−15^ (Mann-Whitney test). (**h**) Cells treated for 24 h with Luciferase- or KLF2-targeting siRNAs were then incubated for 24 h with 2.5 μmol/L simvastatin and processed for immunofluorescence. Scale bar: 20 μm.

One of the mechanistic explanation for the antithrombotic effects of statins is that they up-regulate the expression of Krüppel-like Factor 2 (KLF2)^31^,^35^, a transcription factor known to coordinate a compounded anti-inflammatory and anticoagulant response^43^,^44^. Intriguingly, virus-mediated overexpression of KLF2 in endothelial cells results in the production of mini-WPBs^45^. Due to this link between statins and KLF2, we investigated whether this transcription factor may be responsible for the effects of statins on WPB size. As reported by others^31^,^35^, at all the concentrations we tested, both simvastatin and fluvastatin resulted in KLF2 mRNA up-regulation in HUVECs (Figure S2a) and induction of two of its target genes, vWF (Figure S2b,c) and Thrombomodulin (TM)^46^,^47^ (Figure S2d). KLF2 overexpression was also reported to increase the number of WPBs in endothelial cells^45^. Statin treatment for 24 h had the same effect (Figure S2e,f). Finally, we confirmed that up-regulation of KLF2 results in WPB size shortening^45^, by overexpressing an EGFP-KLF2 fusion protein. Although EGFP-KLF2 expressing cells were fewer than EGFP-expressing controls (Figure S2g,h), HTM analysis at a single-cell level allowed us to identify and separately analyse the WPB population in transiently transfected cells and clearly showed that the size of WPBs was reduced in EGFP-KLF2 positive cells (Fig. 4a,b). Together, these data suggest that KLF2 up-regulation by statins might indeed play a key role in mediating their effects on WPB size. If this is the case, then KLF2 knockdown should prevent the effects of statins on WPB size. Simvastatin and fluvastatin treatment for 24 h increased both KLF2 mRNA and protein levels in Luciferase-siRNA treated cells; this up-regulation was however blocked in KLF2-siRNA cells (Figs 4c and S2i,j). Of note, the increase in WPB numbers due to statin incubation was almost completely blocked by KLF2 knockdown, suggesting that this phenomenon is mediated for the most part by KLF2 up-regulation (Figure S2k). As for WPB size, KLF2 knockdown cells treated with DMSO showed a modest increase in WPB size (Fig. 4d,e and compare insets 3 and 4 to 1 and 2 in 4f). However, upon statin treatment, KLF2 knockdown only partially rescued the WPB size reduction (Fig. 4d,e and compare insets 7 and 8 to 5 and 6 in 4f). This indicates that KLF2 plays only a partial and not a major role in the statin-mediated WPB size reduction. Statins cause a reduction in WPB size through Golgi unlinking (Fig. 2a,f,g). We therefore analysed the effects of KLF2 knockdown on this organelle, finding that it did not rescue the fragmentation caused by statin treatment (Fig. 4g,h). The effects of statin on the Golgi complex thus explain why KLF2 knockdown fails to fully rescue the size of WPBs to control (i.e., Luciferase/DMSO treatment) levels. We conclude that other mechanisms besides KLF2 are responsible for the statin-induced Golgi unlinking and WPB shortening.

### Plasma vWF recruitment to the endothelial surface is sensitive to WPB size

We investigated further the functional consequences of WPB size. *In vitro*, under high wall shear stress conditions, surface immobilized vWF interacts with soluble flowing vWF, promoting its deposition^20^,^48^, a process that *in vivo* may act to amplify the recruitment of platelets and thus growth of platelet aggregates. We therefore tested whether, at physiological venous wall shear stress (2.5 dynes/cm^2^), the secreted endothelial vWF is able to recruit circulating plasma vWF to the cell surface.

**Figure 5:**
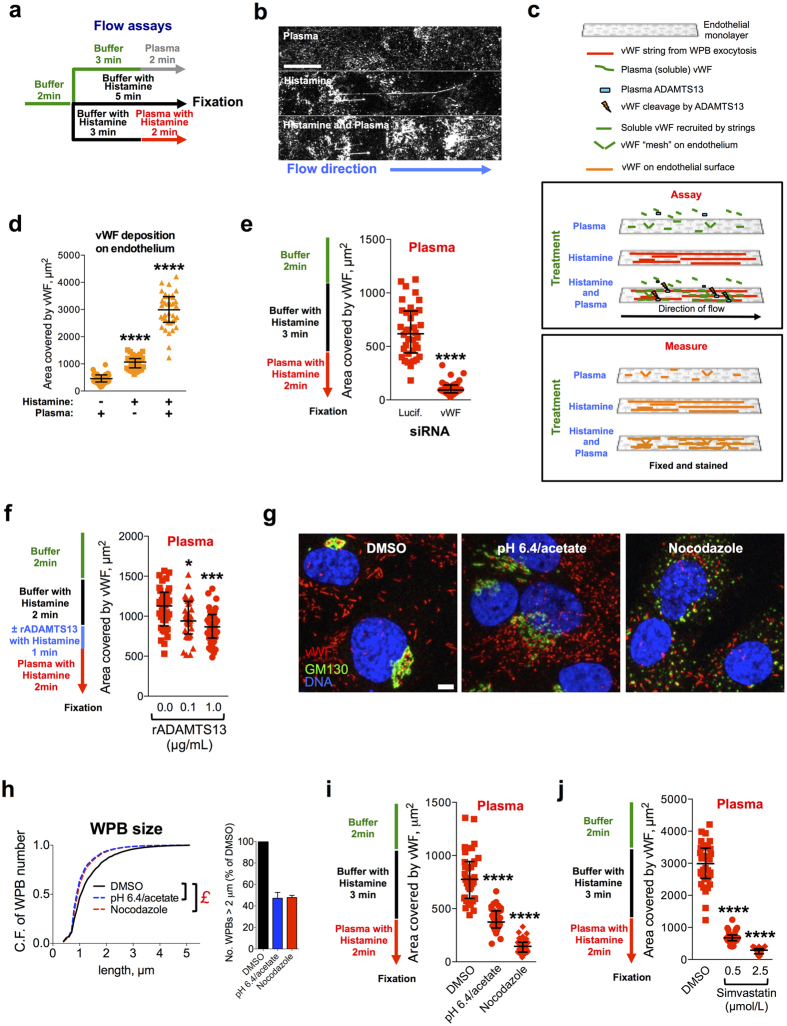
WPB size modulates the recruitment of plasma vWF to the endothelial surface. (**a**) HUVECs were subjected to flow assays as indicated. When WPB exocytosis was stimulated, histamine (100 μmol/L) was supplemented to buffer and human normal pooled plasma. (**b**) At the end of the assays, cells were fixed and the extracellular vWF immuno-stained and imaged. Scale bar: 25 μm. (**c**) Diagram of the procedure in panels (a) and (**b**) illustrates the assay and what it measures. (**d**) vWF associated with the endothelial surface following the assays described in panel (**a**). The area of the vWF signal on the endothelial surface was measured from 36 fields of view (the data points displayed) per condition. Bars: medians and interquartile ranges; histamine stimulation in buffer and histamine stimulation coupled to plasma were compared to the unstimulated plus plasma condition; ****P < 0.0001 (Mann-Whitney test). (**e**) Luciferase- or vWF-siRNA treated HUVECs were subjected to the indicated flow assay (left) and vWF deposition on endothelial cells measured (right); ****P < 0.0001 (Mann-Whitney test). (**f**) vWF plasma recruitment after a brief treatment with recombinant ADAMTS13 (rADAMTS13) at the indicated concentrations; *P < 0.05; ***P < 0.001 (Mann-Whitney test). (**g**) HUVECs treated for 24 h with vehicle (DMSO, control), medium at pH 6.4 and supplemented with acetate or nocodazole (1 μg/mL) were processed for immunofluorescence. Scale bar: 5 μm. (**h**) HTM analysis of HUVECS treated as described in (**g**). Left, Cumulative frequency (C.F.) plots of the total number of WPBs as a function of the organelles’ length. N ~ 10^5^ per condition. £, *P* < 10^−15^ for the indicated pairwise comparisons (Mann-Whitney test). Right; long WPBs (>2 μm). Bars: mean ± range for two replicate HTM experiments. (**i**) HUVECs treated as described and subjected to the indicated flow assay (left) to measure vWF deposition on endothelial cells (right). Quantifications were as described above. ****P < 0.0001 (Mann-Whitney test). (**j**) vWF deposition on HUVECs treated with vehicle (DMSO), 0.5 or 2.5 μmol/L simvastatin for 24 h and perfused as described (left). ****P < 0.0001 (Mann-Whitney test).

We initially tested the effects of human plasma perfusion, to mimic the blood circulation, on endothelial cell monolayers in the absence or presence of histamine, which stimulates WPB exocytosis and vWF secretion (Fig. 5a). Plasma perfusion alone resulted in low amounts of vWF associated with the endothelial surface (Fig. 5b, plasma). Upon histamine stimulation and in the absence of plasma, the vWF secreted by endothelial monolayers adhered to the endothelial surface, most notably as strings (Fig. 5b, histamine). When plasma was perfused concurrently with histamine stimulation, higher levels of surface-adhered vWF were observed, indicating that soluble plasma is recruited to the endothelial surface (Fig. 5b, histamine and plasma). In this condition, fewer and shorter strings were visible, likely because of the proteolytic activity of ADAMTS13 - and possibly of other plasma proteases - which reduce both length and numbers of vWF strings^16^,^19^. The methodology and effects observed in these assays are summarized in the schematic depicted in Fig. 5c. In order to quantify vWF association with endothelial cells and compare treatments, we measured the area covered by its immuno-fluorescent signal. As shown in Fig. 5d, soluble plasma vWF can bind the endothelial surface in the absence of platelets. This recruitment seems to require WPB exocytosis (histamine stimulation), since in un-stimulated cells, even in the presence of plasma, little vWF was associated with endothelial cells (Fig. 5b,d, plasma). Therefore, secretagogue-activated endothelial cells become adhesive to soluble plasma vWF.

To establish whether this recruitment of soluble plasma vWF depends on the secretion of endothelial vWF, we tested the effects of vWF knockdown in this assay. vWF-targeting siRNA treatment of HUVECs resulted in the reduction of both protein levels and WPB numbers^21^ (see also Figure S3a). Surface deposition of secreted endothelial and soluble plasma vWF was abolished when vWF was knocked down (Figs 5e and S3b), indicating that endothelial vWF secretion is required for recruitment of circulating plasma vWF to the cell surface.

To test whether plasma vWF recruitment to the endothelial surface is direct, we carried out experiments with purified plasma vWF (Figure S3c) at the same concentration it has in whole plasma (~10 μg/mL). Perfusion of purified vWF was sufficient to induce an increase in endothelial surface associated vWF signal, but this was not as striking as that seen in the presence of whole plasma (Figure S3d). These data suggest that while plasma vWF can bind directly to the secreted endothelial vWF, other plasma components are likely to co-operate to enhance this interaction.

ADAMTS13 promptly degrades highly multimerized vWF, which, by being more sensitive to shear, exposes its cleavage sites to the protease under flow^6^,^49^. vWF strings are particularly sensitive to ADAMTS13 activity; in its presence their size and number are progressively reduced^16^,^19^. We therefore measured the effects of short treatments with recombinant ADAMTS13 (rADAMTS13) in order to reduce the levels of secreted vWF on the endothelial surface before perfusion with plasma. At concentrations found in plasma of patients with inherited and acquired TTP (0.1 μg/mL)^50^, rADAMTS13 had no effect on vWF associated to the endothelial surface after buffer perfusion (Fig. S3e) and only slightly reduced it when plasma was added (Fig. 5f). Pre-treatment with normal concentrations of rADAMTS13 (1.0 μg/mL), however, resulted in a clear decrease of endothelial-associated vWF in both conditions (Figs 5f and S3e, 1.0 μg/mL rADAMTS13). Together, these results are consistent with the conclusion that secreted endothelial vWF is the relevant factor for the recruitment of plasma vWF to the endothelial surface. Secreted endothelial vWF thus provides adhesive scaffolds that can locally recruit plasma vWF to the endothelial surface. Under flow, the immobilized plasma vWF might transiently (i.e. before ADAMTS13 counteracts this phenomenon) synergize with the vWF strings generated by WPB exocytosis to increase the recruitment of circulating platelets to the endothelial surface.

Our findings imply that mechanisms capable of regulating vWF string size or number can modulate the adhesive properties of a vascular bed towards soluble vWF and platelets. We have shown that one mode of regulation of vWF string length is through control of the size of WPBs. We therefore assessed whether the size of these organelles, in addition to the length of platelet-decorated vWF strings, also influences the capability of its vWF cargo to recruit, when secreted, soluble plasma vWF. To this aim, we prompted formation of mini-WPBs in HUVECs by two experimental treatments that we previously used^21^ to unlink the Golgi ribbon, whose integrity is necessary for the formation of long organelles (Fig. 1a): depolymerisation of microtubules with nocodazole^51^ and incubation with acetate in acidic conditions^21^,^52^ (Figure S3f,g). In both cases, we obtained cells with WPBs that were shorter than controls (Fig. 5g,h). Cells with mini-WPBs showed either no change or a reduced association of secreted vWF with the cell surface, compared to control conditions, when stimulated with buffer containing histamine under flow (Fig. S3h). However, both mini-WPB-producing endothelial monolayers recruited considerably less soluble plasma vWF than controls (Fig. 5i). Of note, upon histamine challenge, one of the mini-WPB-generating treatments (pH 6.4/acetate) released more vWF while the other (nocodazole) released less than control cells (Fig. S3i,j). Thus, the extent of plasma vWF recruitment appears to correlate with the size of WPBs, rather than the total amount of their secreted vWF cargo.

Since statins also reduce WPB size, we tested their effects on the recruitment of soluble plasma vWF to the endothelial surface. We compared controls to cells treated for 24 h with simvastatin at 0.5 and 2.5 μmol/L, concentrations that generate intermediate and maximal effects on mini-WPB production (Fig. 2b–e). At both concentrations, soluble plasma vWF recruitment was dramatically inhibited by statin treatment (Fig. 5j). Thus, consistent with other mini-WPB inducing treatments, statins also reduce plasma vWF recruitment to the endothelium.

## Discussion

Endothelial cells produce Weibel-Palade bodies in a wide range of sizes (0.5–5 μm). Long WPBs contain a substantial fraction of the vWF stored by endothelial cells (Fig. 1b) and may therefore be disproportionately important in defining the activity of the vWF secreted by endothelial cells. In a recent study, we tested this by measuring the size of the platelet-decorated vWF strings generated by control cells or cells enriched in mini-WPBs, observing a positive correlation between WPB size and string length^21^. These findings suggested that endothelial beds may use regulation of WPB size to spatially and temporally modulate their haemostatic propensity as a consequence of physiological cues^21^. This hypothesis predicts that: (a) size control dysregulation, with production of a higher proportion of long WPBs, may contribute to pathological endothelial thrombotic behaviour; (b) such a condition might be ameliorated or reverted by promoting biogenesis of shorter organelles.

The experimental manipulations we used to induce Golgi unlinking and WPB size changes (microtubule depolymerisation, knockdown of Golgi structural proteins, etc.)^21^ do not occur physiologically in cells. Therefore, to validate our hypothesis, we investigated whether pharmacological treatment of endothelial cells was capable of inducing changes in WPB size. We focused on a widely used class of drugs: statins. The rationale for this choice was that, besides lowering cholesterol plasma levels, statins display a wide range of “pleiotropic” effects that are independent from lipid regulation. In particular, statins elicit rapid antithrombotic and anticoagulant responses. Statins decrease pro-thrombotic/pro-coagulant mediators, such as NOX2 (a subunit of NADPH oxidase), Tissue Factor, Plasminogen Activator Inhibitor-1 and PAR-1, while increasing antithrombotic molecules, such as Thrombomodulin, tissue Plasminogen Activator, Tissue Factor Pathway Inhibitor, eNOS and its product nitric oxide^30^,^33^,^34^,^53^. Moreover, statins up-regulate KLF2^31^,^35^, a “master switch” transcription factor that promotes a quiescent, antithrombotic and anti-inflammatory phenotype in endothelial cells^43^,^44^. Interestingly, when overexpressed in endothelial cells, KLF2 induces formation of smaller WPBs^45^, which to the best of our knowledge, is the only experimental evidence indicating the existence of pathways that regulate WPB size.

We found that statin treatment induces a reduction of WPB size that is dependent on Golgi ribbon unlinking, consistent with the current model of WPB biogenesis (Fig. 1a). We also investigated whether KLF2 mediates these statin effects. KLF2 overexpression does, indeed reduce WPB size, and we also show that KLF2 siRNA-mediated depletion slightly increases organelle size. However, upon statin treatment KLF2 depletion, although efficient, is not sufficient for a full rescue of the WPB size to control levels (i.e. as in DMSO-treated Luciferase-siRNA cells). It is the Golgi unlinking induced by statins, and which is not rescued by KLF2 knockdown, that prevents the biogenesis of long WPBs (Fig. 1a) and therefore explains the reason for the failure to rescue size. We must thus conclude that statin-responsive effectors other than KLF2 are involved in the Golgi ribbon unlinking that mediates the reduction of WPB size. What these effectors might be remains to be investigated. The most obvious is the lack of cholesterol, since its depletion in some cell types does result in fragmentation of the Golgi apparatus^54^. However, we cultured cells in medium containing 20% serum in all conditions; it is therefore unlikely that cellular cholesterol is severely depleted even in the presence of statins. Importantly, since statins inhibit HMG-CoA reductase activity and thus block the synthesis of mevalonate and its downstream products, the changes to the Golgi structure might also be the results of signalling pathways sensing the depletion of metabolites other than cholesterol.

It is known that under high wall shear stress, vWF immobilized on a substratum can recruit soluble vWF^48^ and that compared to buffer, at venous shear wall stress, the combined presence of plasma and platelets temporarily increases the size and number of platelet-decorated vWF strings before ADAMTS13 initiates their degradation^20^. We now directly show that, in the absence of platelets, plasma vWF is recruited to the endothelial surface following WPB secretion. This recruitment is dependent on endothelial, highly-multimerized vWF being secreted and assembling into strings, since it is inhibited by both vWF knockdown and recombinant ADAMTS13 pre-treatment. Crucially, WPB size has similar effects to ADAMTS13 treatment, leading to the conclusion that the dimensions of WPBs do affect the homotypic (i.e. towards plasma vWF) adhesive activity of their vWF cargo.

As in the case of other mini-WPB inducing treatments, statin incubation reduced both plasma vWF recruitment and the length of the platelet-decorated vWF strings. Of note, even a small reduction of the fraction of long WPBs, such as that seen at the lowest concentration of statins used in our experiments, was sufficient to elicit an effect on both the homotypic and the heterotypic (towards platelets) adhesive activity of the secreted vWF. This seems to indicate that functional effects become apparent when the fraction of long WPBs falls below a threshold.

How WPB size regulates the length of the strings generated by vWF secretion is at present unclear. It is known that vWF multimerization can affect string length^40^. However, we show here that in short WPBs, induced by either statin or nocodazole treatment, multimerization is not impaired and therefore cannot account for the difference in size of the platelet-decorated vWF strings that are generated. We conclude that the size of the organelle, imparting an as yet unknown spatial arrangement to the packaged vWF, results in the formation of strings of differing length.

Statins reduce vWF secretion from endothelial cells^55^,^56^. Could this reduction in vWF release explain the effects on plasma vWF recruitment to the endothelial surface and on the length of platelet-decorated vWF strings observed when cells are treated with statins? While this possibility cannot be ruled out, our results suggest otherwise. First, we found that rather than the total amount of vWF released it is the size of WPBs that correlates best with the recruitment of plasma vWF to the endothelial surface (Figs 5i and S3i). Second, independently of the number of the platelet-decorated vWF strings generated at the cell surface, statin treatment does indeed affect their lengths (Fig. 3b).

Statins’ effect on WPB size is rapid. Interestingly, as shown in animal and clinical studies, the antithrombotic response elicited by statins also have a fast onset that is unexplained by their effects on plasma cholesterol^29^,^30^,^32^,^33^,^34^,^57^,^58^. It is therefore tantalizing to suggest, based on the results we present, that statins’ effect on WPB size could contribute to the overall antithrombotic phenotype induced by these drugs. However, whether our *in vitro* findings regarding the statin effects on WPB size can be translated to *in vivo* models and clinical settings remains to be tested experimentally.

**Figure 6:**
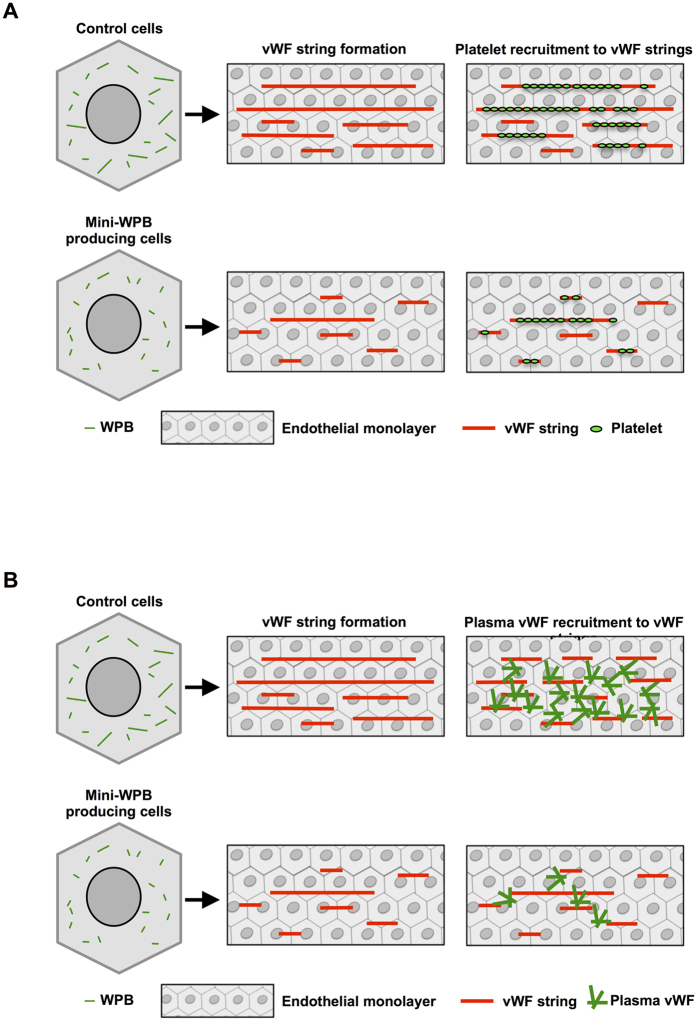
Functional consequences of WPB size. (**A**) Cultured endothelial cells produce WPBs with lengths ranging between 0.5 and 5 μm. Under flow, which mimics blood circulation, WPB exocytosis releases highly multimerized vWF generating vWF strings, which function as adhesive platforms for the recruitment of platelets on the endothelial surface. In mini-WPB producing cells, vWF exocytosis generates shorter platelet-decorated vWF strings. (**B**) Exocytosis of mini-WPBs, such as those produced by treatment with statins, deploys vWF that is less active in recruiting the soluble vWF present in circulating plasma. This diminished self-adhesion may synergize with the reduced platelet recruitment.

Nonetheless, this study represents a proof of concept. By confirming and significantly expanding our previous observations, the present findings provide further support to the notion that WPB size plays a role in modulating the adhesive activity of its vWF cargo. Since plasma vWF is required for platelet aggregation^48^, control of WPB size, by affecting the recruitment of both these factors, may represent a regulatory node for platelet aggregation to the endothelial cell surface (Fig. 6). Considering that signalling pathways that affect the integrity of the Golgi ribbon do exist^59^, the physiological modulation of WPB size (Fig. 1a) is not only possible but indeed likely. Compounds capable of intercepting these pathways, whether they will turn out to be statins or others, may thus represent new tools in the treatment of thrombotic pathologies.

## Materials and Methods

### Cells

Human umbilical vein endothelial cells (HUVECs) pooled from donors of both sexes were commercially obtained from Lonza or PromoCell. Cells were used within passage 5 (~15 population doublings since isolation from umbilical cord).

### Antibodies and reagents

Four antibodies to vWF were used: a rabbit polyclonal antibody to vWF pro-peptide region, a kind gift by Dr. Carter (St. George’s University, London), was previously described^60^; a rabbit polyclonal antibody from DAKO (cat. no. A0082); a sheep polyclonal antibody from AbD Serotec (cat. no. AHP062); and a HRP-conjugated rabbit polyclonal antibody from DAKO (cat. no. P0226). The antibody to pan-Tubulin (sheep polyclonal, cat. no. ATN02) was from Cytoskeleton. Anti-Thrombomodulin (mouse monoclonal, D-3) antibody was from Santa Cruz biotechnology. Mouse monoclonal antibodies to GM130, VE-Cadherin, and γ-Adaptin were from BD Bioscience. Anti-β3 integrin antibody (rabbit polyclonal, cat. no. 4702) was from Cell Signalling Technology. Anti-GPIIb/CD41 (clone 5B12) mouse monoclonal antibody was from Millipore. Anti-KLF2 raabbit polyclonal antibody was from Novus Biologicals (cat. no. NBP2-31619). Histamine was either from Sigma-Aldrich (cat. no. H7250) or Enzo Life Sciences (cat. no. ALX-550-132). Tyrode salts (T2145), nocodazole (M1404), simvastatin (S6196) and fluvastatin sodium hydrate (SML0038) were all from Sigma-Aldrich. Human recombinant full-length ADAMTS13 was from R&D Systems (cat. no. 6156-AD-020). Purified plasma vWF was kindly provided by Dr. Mckinnon and Prof. Laffan (Imperial College London). The plasmids used in this study are described in the Supplementary Information.

### WPB secretion

WPB secretion was stimulated by challenging HUVECs with histamine at a final concentration of 100 μmol/L.

### High-throughput morphometry (HTM)

Image processing, organelle segmentation and measurement of morphological parameters (high-throughput morphometry, HTM) have been described in detail elsewhere^21^. Briefly, images of the organelle channel (i.e., WPBs or Golgi) were processed with ImageJ using the “rolling ball” background subtraction and then subjected to segmentation with an auto local threshold, the “Bernsen’s method” (https://fiji.sc). This high-throughput imaging approach provides an unbiased, deep probing of samples, detecting quantitative morphological differences with a high level of confidence. Details of the HTM analysis of EGFP and EGFP-KLF2 expressing cells are provided in the Supplementary Information.

### Flow assays

HUVECs seeded on Ibidi μ-slides were maintained at 37 °C and stimulated to secrete vWF while perfused with buffer, normal human pooled plasma or washed platelets at a constant wall shear stress of 0.25 MPa (2.5 dynes/cm^2^). Details are available in the Supplementary Information.

### Statistical analysis

Non-parametric, two-tailed, two independent sample Mann-Whitney test was used, except when explicitly stated otherwise. *R* (http://www.r-project.org/) was used to analyse HTM datasets. In all other cases, we used Prism 6 (GraphPad) statistical package.

## Additional Information

**How to cite this article**: Ferraro, F. *et al*. Weibel-Palade body size modulates the adhesive activity of its von Willebrand Factor cargo in cultured endothelial cells. *Sci. Rep.* **6**, 32473; doi: 10.1038/srep32473 (2016).

## Supplementary Material

Supplementary Information
