# Erratum: Weibel-Palade body size modulates the adhesive activity of its von Willebrand Factor cargo in cultured endothelial cells

**DOI:** 10.1038/srep33938

**Published:** 2016-09-26

**Authors:** Francesco Ferraro, Mafalda Lopes da Silva, William Grimes, Hwee Kuan Lee, Robin Ketteler, Janos Kriston-Vizi, Daniel F. Cutler

Scientific Reports 6: Article number: 3247310.1038/srep32473; published online: 08 31 2016; updated: 09 26 2016.

The original version of this Article contained a typographical error in the spelling of the author Mafalda Lopes da Silva which was incorrectly given as Silva Mafalda Lopes da. This has now been corrected in the PDF and HTML versions of the Article.

