# Supplementary material for: Weibel-Palade body size modulates the adhesive activity of its von Willebrand Factor cargo in cultured endothelial cells
Source: Sci Rep. 2016 Aug 31;6:32473. doi: 10.1038/srep32473 (PMC5006059; doi:10.1038/srep32473)

Supplementary information

Weibel-Palade body size modulates the adhesive activity of its von Willebrand Factor cargo in cultured endothelial cells

Francesco Ferraro[1]*, Mafalda Lopes da Silva[1], William Grimes[1,2], Hwee Kuan Lee[2], Robin Ketteler[1], Janos Kriston-Vizi[1], and Daniel F. Cutler[1]*

Affiliations

[1]MRC Laboratory for Molecular Cell Biology, UCL, Gower Street, London, WC1E 6BT

[2]Imaging Informatics Division, Bioinformatics Institute, A*STAR 30 Biopolis Street #07-01, Matrix, Singapore 138671

*** Corresponding authors**

Francesco Ferraro
MRC Laboratory for Molecular Cell Biology, UCL, Gower Street, London, WC1E 6BT, United Kingdom
Email: f.ferraro@ucl.ac.uk
Phone: +44 (0) 20 7679 7903

Daniel F. Cutler
MRC Laboratory for Molecular Cell Biology, UCL, Gower Street, London, WC1E 6BT, United Kingdom
Email: d.cutler@ucl.ac.uk
Phone: +44 (0) 20 7679 7808

Contains Supplementary Materials and Methods, Supplementary Figures S1, S2 and S3 and Supplementary references.

Supplementary Materials and Methods

Cells. Primary endothelial cells (human umbilical vein endothelial cells, HUVECs were expanded in our laboratory. Cells were maintained in HUVEC Growth Medium (HGM): M199 (Gibco, Life Technologies) supplemented with 20% Fetal Bovine Serum, (Labtech), 30 µg/mL endothelial cell growth supplement from bovine neural tissue and 10 U/mL Heparin (both from Sigma-Aldrich). Cells were used within passage 5 (~ 15 population doublings since isolation from umbilical cords) and treatments were carried out with buffers, supplements and drugs diluted in HGM.

Golgi ribbon unlinking. To unlink the Golgi ribbon, HUVECs were either incubated in HGM supplemented with nocodazole (1 µg/mL final concentration) or with 30 mmol/L 2-(N-Morpholino)ethanesulphonic acid (MES) and 25 mmol/L sodium acetate (both from 1 mol/L stocks). The pH of the growth medium supplemented with MES/acetate was 6.4 [1, 2]. DMSO added to HGM (final 0.01%) was used as a control treatment.

Statin treatment. Simvastatin and fluvastatin treatments were carried out for 24 h. HUVECs were fed HGM with the compounds diluted to final concentrations of 0.5, 2.5 and 12.5 µmol/L. DMSO (0.05% final concentration) and nocodazole (1 µg/mL final concentration) dilutions in HGM were used as control treatments. Stock solutions of simvastatin and fluvastatin were made in DMSO at 25 mmol/L, stored at – 20 °C and used within 6 months.

Confocal microscopy. Confluent HUVECs grown on gelatin-coated coverslips or 96well plates (Nuclon surface©, NUNC) were treated as indicated and then fixed in 4% formaldehyde in PBS for 10 min at RT. Fixed cells were permeabilised with 0.2% TX-100 in PBS for 10 min at RT and then incubated with 5% bovine serum albumin (BSA) in PBS. Samples were then incubated with primary antibodies and then secondary antibodies (conjugated to Alexa Fluor dyes, Life Technologies) diluted in 0.02% TX-100/1% BSA in PBS. Coverslips were mounted with ProLong Gold antifade reagent (Life

Technologies), while samples in 96well plates were kept in PBS. Samples were imaged with 40x (NA 1.15) or 63x (NA 1.3) oil immersion objectives on a Leica Microsystems TCS SPE3 confocal system. Micrographs shown in the figures are maximum intensity projections of image stacks. For high-throughput confocal microscopy, samples in 96 well plates were imaged with an Opera High Content Screening System (Perkin Elmer), using the 40x air objective (NA 0.6). Nine to 20 fields of view per well were acquired, allowing visualization of tens of thousands of WPBs and Golgi elements. Cells grown on Ibidi µ-slides for flow assays were fixed under flow. In order to selectively visualize extracellular vWF, in this case permeabilisation was omitted. Cells were blocked and incubated with primary and fluorophore-conjugated secondary antibodies as described above. Image acquisition was with carried out with a 40x (NA 1.15) oil immersion objective on a Leica Microsystems TCS SPE3 confocal system. Contiguous fields of view (for a total of 36, in a 3 x 12 arrangement) were imaged. The fields of view were analyzed independently in the case of buffer and plasma perfusions or a composite tile in platelet flow assays.

Quantification of the number of WPBs per cell. The number of WPBs per cell was calculated from HTM experiments on a well basis by measuring the number of organelles and dividing it by the number of nuclei. Therefore, for this parameter the number of data points corresponded to the number of wells per treatment in each experiment.

HTM of WPBs in EGFP and EGFP-KLF2 expressing cell. HUVECs nucleofected with plasmids encoding EGFP and EGFP-KLF2 were seeded on 96well plates as described for other treatments, with the exception of their density (20000 cells per well instead of 15000). Cells were cultured for 24 h in HGM and then fixed. Cells were immuno-stained to label WPBs and VE-cadherin and counterstained with Hoechst 33342 ((Life Technologies) to label the nuclei. Due to the low efficiency of expression of the EGFP-KLF2 fusion protein, fifty fields of view per well were acquired. In order to identify EGFP-positive cells, an automated workflow was designed. A mask for all nuclei was created and applied to the EGFP channel. A threshold was established for the

EGFP signal and cells with values above the threshold were identified as expressing the exogenous proteins (EGFP or EGFP-KLF2). Morphometric analysis of the WPBs within EGFP-positive cells was carried out after identification of cell boundaries with the VE-cadherin signal.

Quantification of Golgi fragmentation from low-throughput confocal imaging. Image series acquired on a Leica confocal systems were opened in ImageJ and the Golgi channel was segmented using a fixed global threshold for all the treatments. Binary images were then analyzed using the "analyze particle" tools with a cut off for the area size (excluding objects < 0.1 μm^2).

Western blotting. Cell lysates were prepared on ice using cold RIPA buffer (100 mmol/L Tris-HCl, pH 7.5, 150 mol/L NaCl, 1% TX-100, 0.5% Na-Deoxycholate and 0.05% SDS) supplemented with protease inhibitors (Sigma-Aldrich) and clarified by centrifugation. For KLF2quantification, whole cell lysates were prepared in SDS-lysis buffer (0.5% SDS, 2 mmol/L EDTA, 50 mmol/L NaF, 50 mmol/L Tris, pH 8.0), supplemented with protease inhibitors and passed through a needle 10 times to shred the DNA. Protein concentration in lysates was measured with the Pierce BCA Protein Assay Kit (Thermo Scientific). Equal amounts of lysates, by protein, were denatured in reducing buffer and fractionated by SDS-PAGE and electro-blotted on PVDF membranes (Millipore). After blocking with 5% BSA in 0.05% Twin-20 PBS (PBS-T), membranes were incubated with antibodies diluted in 5% BSA/PBS-T and then with the appropriate HRP-conjugated secondary antibodies (Jackson Immunoresearch Laboratories). ECL signals generated using the Luminata™ Crescendo Western HRP-substrate (Millipore) were digitally acquired with an ImageQuant LAS 4000 CCD (GE Healthcare Life Sciences) and quantified with ImageJ.

vWF ELISA. vWF content in cell lysates, releasates and subcellular fractions was measured using a sandwich ELISA. Maxisorp (NUNC) 96well microtiter plates were coated with anti-human vWF antibody (DAKO, cat. no. A0082) diluted (1:600) in PBS for 1 h. Blocking was carried out for 1 h with buffer 2xTBE [1% TX-100, 0.4% fish skin gelatine (Sigma-Aldrich, cat. No. G7765),

1 mmol/L Na_2-EDTA in PBS]. Each sample was applied to duplicate wells and incubated for 1h, followed by washes in 1xTBE (2xTBE diluted with 1 volume of PBS). HRP-conjugated anti-human vWF (DAKO, cat. no. P0226) diluted (1:1000) in 1xTBE was then applied and incubated for 1 h, followed by four rinses in 2x TBE and one in PBS. O-phenyldiamine, (Sigma-Aldrich, P9029; saturated solution in methanol) and H_2O_2 (30% solution in water, Sigma-Aldrich, H3410) diluted to final concentrations of 0.6% and 0.03%, respectively, in sodium citrate/sodium phosphate monobasic solution, pH 6.5 were used to measure HRP activity. Kinetic assays (30 min) were carried out by reading the absorbance at 450 nm with a VersaMax plate reader (Molecular Devices). vWF amounts were calculated with the SoftMax software (Molecular Devices) using standard curves generated by serial dilution of human pooled plasma (Sigma-Aldrich, P9523). vWF absolute amounts were calculated by converting microliters of pooled plasma used for standard curves into ng of vWF (1 µL plasma contains 10 ng vWF).

sIRNAs. siRNA were custom synthesized (Eurofins MWG Operon). Targets and siRNA sequences:
Firefly Luciferase, sense, 5'-CGUACGCGGAAUACUUCGA[dT][dT]-3';
hvWF, sense: 5'-GGGCUCGAGUGUACCAAAA[dT][dT]-3';
hKLF2-1, sense: 5'-GCACCGACGACGACCUCAA[dT][dT]-3';
hKLF2-3, sense: 5'-UGCUGGAGGCCAAGCCAAA[dT][dT]-3';
hKLF2-4, sense: 5'-CCAAGAGUUCGCAUCUGAA[dT][dT]-3'.
All siRNAs were described and validated in other studies [3-6]. siRNAs were delivered to cells using an AMAXA Nucleofector II (Lonza). A constant number of HUVECs was used per reaction (2 millions). Cells treated with siRNAs targeting vWF and its Luciferase control were delivered at 500 pmol per reaction and analysed at 48 h. siRNAs targeting KLF2 were pooled and delivered at 500 pmol each per reaction; controls were in this case nucleofected with Luciferase- targeting siRNA at 1500 pmol per reaction. Twenty-four h post-nucleofection, cells were incubated with DMSO or 2.5 µmol/L simvastatin. Cells were lysed for qPCR processing or fixed for HTM analysis after 24 h (i.e., after 48 h of siRNA treatment).

Plasmids. The vWF-GFP plasmid was described previously [7]. EGFP-KLF2 coding sequence was inserted in the pCineo vector, using primers designed by the NEBuilder online tool (http://nebuilder.neb.com/). An amplicon encoding EGFP with flanking sequences homologous to pCiNeo and GlyGlyGlySerGlyGlyGlySer (linker)-KLF was generated by PCR with the pEGFP-N1 vector as a template and the following primers:
forward, ttaatacgactcactataggctagcATGGTGAGCAAGGGCGAG
(pCineo sequence: lower case; EGFP sequence: upper case);
reverse, acctgatccaccgccCTTGTACAGCTCGTCCATGC
(GlyGlyGlySerGlyGlyGlySer linker sequence: lower case; EGFP sequence; upper case). The following primers were used to generate by PCR an amplicon encoding GlyGlyGlySerGlyGlyGlySer linker-KLF2 with flanking regions homologous to the EGFP and the pCineo sequences:
forward, gctgtacaag*ggcggtggatcaggtggaggatct*ATGGCGCTGAGTGAACCC
(EGFP sequence: lower case; GlyGlyGlySerGlyGlyGlySer linker sequence: italics; KLF2 sequence: upper case);
reverse, ctctagaggtaccacgcgtgaattcCTACATGTGCCGTTTCATGTG (pCineo sequence: lower case; KLF2 sequence: upper case). The pCMV6 KLF2-Myc-DDK vector (Origene, cat. no. RC210042), encoding human KLF2, was used as a template. Amplifications were carried out with Q5 High-Fidelity DNA Polymerase (NEB, cat. no. M0491). The pCineo vector, linearized by digestion with NheI and EcoRI, and the amplicons were assembled with the NEBuilder HiFi DNA assembly cloning kit (New England Biolabs, cat. no. E5520). The correct construct was verified by sequencing.

Plasmids were delivered into HUVECs as described for siRNAs. Reactions were carried out with 2 million cells and 7.5 µg of vWF-GFP plasmid or 2 pmol of the pCineo KLF2-EGFP plasmid and of its control, pEGFP-N1. Cells were fixed, stained as described, and analyzed at 24 h post-nucleofection.

Flow assays with buffer and plasma. HUVECs were seeded on gelatin-coated µ-slides I or VI (Ibidi) at a density resulting in confluent monolayers after 24 h and then treatments were started and carried out for 24 h before initiating the assays. Alternatively, untreated cells were cultured for 48 before

the flow assays. µ-slides were connected to a pump system (Harvard Apparatus, Holliston, MA, USA) under temperature control (37 °C). Hanks' balanced salt solution (HBSS, Life Technologies), containing Ca^{2+}, Mg^{2+} and supplemented with 0.2% BSA, was perfused for 2 minutes to rinse out the growth medium and loosely attached cells. The same buffer, supplemented with 100 µmol/L histamine to stimulate WPB exocytosis, was perfused for 3 min, followed by either the same buffer with histamine or normal pooled human plasma (Cryochek™, cat. no. CCN-15, Precision Biologic) supplemented with histamine for two more min. Buffer and plasma were pre-warmed at 37 °C. A constant wall shear stress of 2.5 dynes/cm^2 (0.25 MPa) was maintained throughout the experiments. Finally, cells were fixed under flow with 4% formaldehyde in PBS for 5 min, gradually reducing the flow rate to zero and were left in fixative for 5 more min before being rinsed in PBS. In some experiments, cells were incubated in flowing buffer supplemented with histamine and recombinant ADAMTS13 as the described concentrations for 1 min before final perfusion with either buffer or plasma. Samples were processed for immunofluorescence, without a permeabilisation step, to visualize extracellular vWF and DAPI (Sigma-Aldrich) or Hoechst 33342 (Life Technologies) were used to counterstain nuclei. Stacks of confocal images were acquired with constant parameters for all the conditions. Maximum intensity projection images of the vWF channel in all conditions were segmented in ImageJ using a global threshold. The resulting binary images were then analyzed with the "analyze particles' tool to measure areas.

Preparation of washed platelets. Citrated blood from healthy, non-medicated donors was centrifuged at 250 g for 16 min. Platelet-rich plasma (PRP) was recovered and platelets counted with a hemocytometer. Platelets were separated from plasma by centrifugation and re-suspended in BSA (0.2%) containing HEPES-buffered Tyrode solution, supplemented with 0.02 U/mL apyrase, 10 µmol/L indomethacin (both from Sigma-Aldrich) and 140 nmol/L PGE1 (Enzo Life Sciences) to inhibit activation. Re-suspended platelets were left at room temperature for 10 min and then subjected to two more centrifugations and re-suspensions in Hepes-Tyrode solution with

inhibitors and left at room temperature until used. Immediately before perfusion, washed platelets were centrifuged again and re-suspended in warm (37 °C) Hanks' balanced salt solution (HBSS, containing Ca^{2+}, Mg^{2+} and 0.2% BSA) supplemented with 100 µmol/L histamine.

Platelet-decorated VWF strings. Experiments were performed essentially as described for buffer/plasma perfusions with a few modifications. After initial perfusion with HBSS devoid of histamine, cells were then subjected to perfusion with HSBB supplemented with 100 µmol/L histamine for 2 min, followed by washed platelets at a concentration of 0.5-1 x 10^8 per mL in HSBB containing histamine for 5 min. Phase contrast video-microscopy was carried out during perfusions. Flow rate was adjusted to generate a wall shear stress of 0.25 MPa (2.5 dynes/cm^2) was used throughout the experiments. Cells were then fixed under flow with 4% formaldehyde in PBS for 5 min, gradually reducing the flow rate to zero. Samples were left in fixative in static conditions for 15 more min to minimize platelet dislodgement from the endothelial surface during immuno-staining. Extracellular vWF (to visualize strings) and CD41 (to visualize platelets) were immuno-labeled and DAPI (Sigma-Aldrich) or Hoechst 33342 (Life Technologies) were used to counterstain nuclei. Stacks of confocal images were acquired as described and combined to generate tilescans. Maximum intensity projections of the tilescans were used for quantification of length with ImageJ, using the "straight line" tool. Platelet string length was defined as the maximum distance between platelets decorating an underlying vWF string.

Preparation of cell-free WPBs. Subcellular fractions containing cell-free WPBs were generated essentially as described [2]. Briefly, HUVECs treated as indicated were homogenized on ice in 250 mmol/L sucrose, 5 mmol/L Na_2-EDTA, 20 mmol/L HEPES-NaOH, pH 7.4, supplemented with protease inhibitor cocktail (homogenization buffer, HB). Homogenates were centrifuged at 1000 g for 5 min (at 4 °C). The post-nuclear supernatants thus generated were loaded on two Nycodenz (Axis-Shields) cushions (15% and 40% Nycodenz, both in HB) and centrifuged at 18000 x g and 4 °C for 30 min with

MLS50 (Beckman Coulter) in an Optima™ Max ultracentrifuge. The WPB-containing fractions were recovered at the interface between the nycodenz cushions.

vWF multimer analysis. For vWF multimer analysis, WPB-containing subcellular fractions, were processed as follows. Sample volumes with similar vWF content, as measured by ELISA, were supplemented with 0.4 volumes of 20% SDS and 0.35 volumes of 5x non-reducing SDS-PAGE sample buffer. The samples were incubated for 10 min at 80 °C and then for 30 min at 60 °C. Human plasma was treated as described for the cellular fractions or, to reduce multimers to monomers, with the same reagents supplemented with dithiothreitol (DTT) at a final concentration of 10 mmol/L and incubated for 10 min at 80 °C. Before incubation at 60 °C, N-ethylmaleimide (NEM, 25 mmol/L final concentration) was added to block free thiols and quench residual DTT. The samples were then fractionated by electrophoresis on agarose gels (1.4% high gelling temperature agarose, Lonza) prepared as described [8] at constant current (20 mA). Gels were then incubated at room temperature for 30 min with 1% β-mercaptoethanol in SDS-PAGE running buffer to reduce disulfide bonds and facilitate transfer of large-multimer vWF onto PVDF. Transfer was at constant voltage (16 V) for 16 h at 4 °C. vWF multimers were detected using an HRP-conjugated anti-vWF.

vWF Secretion assay. The amounts of vWF released by HUVECs in 30 min at 37 ºC in serum free medium (M199 supplemented with 10 mmol/L HEPES-NaOH, pH 7.4 and 0.1 mg/mL BSA) in the absence or the presence of secretagogue (histamine, 100 µmol/L) were quantified by ELISA and expressed as percent of total vWF content (obtained by adding the amounts of VWF in lysates and releasates) or as ng of vWF per µg of lysate protein. Since basal release of vWF occurs continuously, total vWF cell content in samples from secretion assays was calculated as: (vWF in lysate + vWF in stimulated releasate – vWF in basal releasate)/lysate protein.

vWF filaments generated from cell-free WPBs. Subcellular fractions containing WPBs (approximately equal vWF amounts by ELISA) were serially diluted in 96-well Maxisorp™ plates (NUNC, Thermo Scientific) and centrifuged (2000 rpm, GH3.8 rotor Beckman Coulter) for 15 min at 4 °C. Adsorbed membranes were permeabilised (30 min on ice with 1% Triton X-100 in homogenization buffer) and fixed with 4% formaldehyde in the same buffer. Samples were immuno-stained for vWF. vWF filaments were imaged by wide-field microscopy with a 20x objective lens (NA 0.4) on a Leica DM IRB microscope.

Quantitative PCR. RNA was prepared from treated HUVECs using an RNeasy kit (Qiagen), and equal amounts of RNA were used to prepare cDNA using the SuperScript III first-strand synthesis system (Life technologies). The following primers were used:
VWF forward, 5′-GCCATCATGCATGAGGTCAGA-3′;
reverse, 5′-GGCTCCGTTCTCATCACAGAT-3′;
actin forward, 5′-TGGTGGTGAAGCTGTAGCC-3′;
reverse, 5′-GCGAGAAGATGACCCAGAT-3′.
QuantiTect Primer assay Hs-KLF2_1_SG (Qiagen, cat. no. QT00204729) was used for human KLF2. DNA amplification was monitored by incorporation of SYBR green (DyNAmo SYBR Green qPCR Kit, Thermo Scientific) and analyzed on a Mastercycler ep Realplex thermocycler (Eppendorf). Gene expression was assessed using the ΔΔCT method and normalized to actin**.** Single product amplification by the specific primers was verified by agarose gel electrophoresis.

Supplementary Figure Legends

Supplementary Figure 1. (**a**) Micrographs of HUVECs grown for 24 h in medium containing vehicle (DMSO), statins or nocodazole at the indicated concentrations. Wider fields of view than in Figure 2A and all the treatments are shown. Scale bar: 20 µm. (**b**) Unlike nocodazole, Golgi ribbon fragmentation induced by statins does not depend on depolymerisation of microtubules. HUVECs treated as indicated were immuno-stained with antibodies to VE-cadherin (to label cell boundaries) and tubulin. Fluvastatin (not shown) had the same effect as simvastatin. Scale bar: 25 µm.

Supplementary Figure 2. (**a**-**c**) Simvastatin and fluvastatin concentration-dependently induce KLF2 and vWF transcription and vWF translation. Quantifications were from triplicate samples (mean ± S.D.). Triangles represent increasing concentrations of statins (0.5, 2.5 and 12.5 µmol/L). (**d**) Expression in HUVECs of Thrombomodulin (TM), one of KLF2 target genes, was increased in a concentration-dependent fashion by statins. **, $P < 0.01$ (or lower; not shown for simplicity) for all treatments compared to DMSO control (Student's t-test). (**e**-**f**) Statin treatment increases the number of WPBs in HUVECs; data points represent 16 determinations per treatment; bars: medians and interquartile ranges. **, $P < 0.01$; ***, $P < 0.001$; ****; $P < 0.0001$ (Mann-Whitney test). (**g**) Expression efficiency of EGFP and EGFP-KLF2 in HUVECs; mean values ± SD of 16 measurements by HTM are shown. (**h**) EGFP-KLF2 localizes to the nucleus (asterisks) of expressing cells and cross-reacts with anti-KLF2 antibody. Scale: 10 µm. (**i**) KLF2-targeting siRNAs efficiently reduce KLF2 transcription in vehicle (DMSO) and simvastatin (2.5 µmol/L) treated cells (24 h). Means ± S.E.M. of three experiments. *, $P < 0.05$; ****; $P < 0.0001$ (Student's t-test). (**j**) KLF2 protein up-regulation by simvastatin and fluvastatin (2.5 µmol/L for 24 h) is blocked by KLF2 knockdown. Scale, 20 µm. (**k**) KLF2 knockdown blocks almost completely the increase of WPB numbers by simvastatin (2.5 µmol/L for 24 h). Quantifications were done as described in panel (**e**) and (**f**). *; $P < 0.05$; ****; $P < 0.0001$ (Mann-Whitney test).

Supplementary Figure 3. (**a**) Left; vWF cell content in Luciferase- (control) and vWF-siRNA treated HUVECs at 48 h post-nucleofection was measured by ELISA and normalized to lysate protein. Mean ± SD from triplicate samples from a nucleofection experiment. Right; Luciferase- or vWF-siRNA treated HUVECs were processed for immunofluorescence of the indicated markers. Bottom, three magnified cells per treatment are shown. Scale bar: 20 µm. (**b**) Cells treated with the indicated siRNAs for 48 h were subjected to the indicated flow assay (left) and the vWF associated with the endothelial surface measured (right) as described in Figure 5. ****, $P < 0.0001$ (Mann-Whitney test). (**c**) Aliquots of one µg of purified plasma vWF were fractionated by SDS-PAGE, transferred to a PVDF and stained with coomassie brilliant blue (CBB, left) or probed for western blot (anti-vWF, right). (**d**) Recruitment to stimulated endothelial cells of soluble purified plasma vWF and soluble vWF from whole plasma was compared using the assay described (left). Purified vWF was used at the concentration present in pooled normal plasma (10 µg/mL). *, $P < 0.05$; ****, $P < 0.0001$ (Mann-Whitney test). (**e**) Untreated HUVECs were subjected to the described flow assays (left) to test the effects of a short treatment with rADAMTS13 at the concentrations indicated. *, $P < 0.05$ (Mann-Whitney test). (**f**) Cells treated for 24 h as indicated were incubated on ice (a condition that induces microtubule depolymerisation) for 60 min and then fixed and immuno-stained to label the Golgi complex (GM130). At low temperature and in the absence of microtubules, the membrane continuity of the intact Golgi ribbons acts against fragmentation (DMSO). In already unlinked Golgi complexes, disruption of microtubules by incubation on ice shows more clearly the separated fragments (pH 6.4/acetate). Nocodazole is shown for comparison. Bottom, three magnified cells per treatment are shown. Scale bar: 20 µm. (**g**) For the samples shown in panel (**f**), the size of GM130-positive objects was quantitatively analyzed from series of confocal images. The fraction of Golgi objects ≥ 10 μm^2 was used as a measure of the extent of Golgi fragmentation in each treatment. (**h**) Control- and mini-WPBs producing cells were subjected to the indicated flow assay (left) and the vWF associated with the endothelial surface measured (right). ****, $P < 0.0001$ (Mann-Whitney test). (**i**) vWF secreted upon histamine challenge (100 µmol/L for 30 min) from cells treated as described was

measured by ELISA and normalized to lysate protein content. (**j**) vWF cell content was measured by ELISA, normalized to protein and expressed as % of DMSO control. In (**i**) and (**j**), data were from 9 determinations (from 3 independent experiments). ** *P* < 0.01; ***, *P* < 0.001 (Mann-Whitney test).

Supplementary References

1. Yoshida, T., Kamiya, T., Imanaka-Yoshida, K. & Sakakura, T. Low cytoplasmic pH causes fragmentation and dispersal of the Golgi apparatus in human hepatoma cells. *International journal of experimental pathology* **80**, 51-57 (1999).

2. Ferraro, F. *et al.* A two-tier Golgi-based control of organelle size underpins the functional plasticity of endothelial cells. *Developmental cell* **29**, 292-304 (2014).

3. Elbashir, S.M. *et al.* Duplexes of 21-nucleotide RNAs mediate RNA interference in cultured mammalian cells. *Nature* **411**, 494-498 (2001).

4. Starke, R.D. *et al.* Endothelial von Willebrand factor regulates angiogenesis. *Blood* **117**, 1071-1080 (2011).

5. Sen-Banerjee, S. *et al.* Kruppel-like factor 2 as a novel mediator of statin effects in endothelial cells. *Circulation* **112**, 720-726 (2005).

6. Parmar, K.M. *et al.* Statins exert endothelial atheroprotective effects via the KLF2 transcription factor. *The Journal of biological chemistry* **280**, 26714-26719 (2005).

7. Romani de Wit, T., Rondaij, M.G., Hordijk, P.L., Voorberg, J. & van Mourik, J.A. Real-time imaging of the dynamics and secretory behavior of Weibel-Palade bodies. *Arteriosclerosis, thrombosis, and vascular biology* **23**, 755-761 (2003).

8. Nightingale, T.D., Pattni, K., Hume, A.N., Seabra, M.C. & Cutler, D.F. Rab27a and MyRIP regulate the amount and multimeric state of VWF released from endothelial cells. *Blood* **113**, 5010-5018 (2009).

Supplementary Figure 1

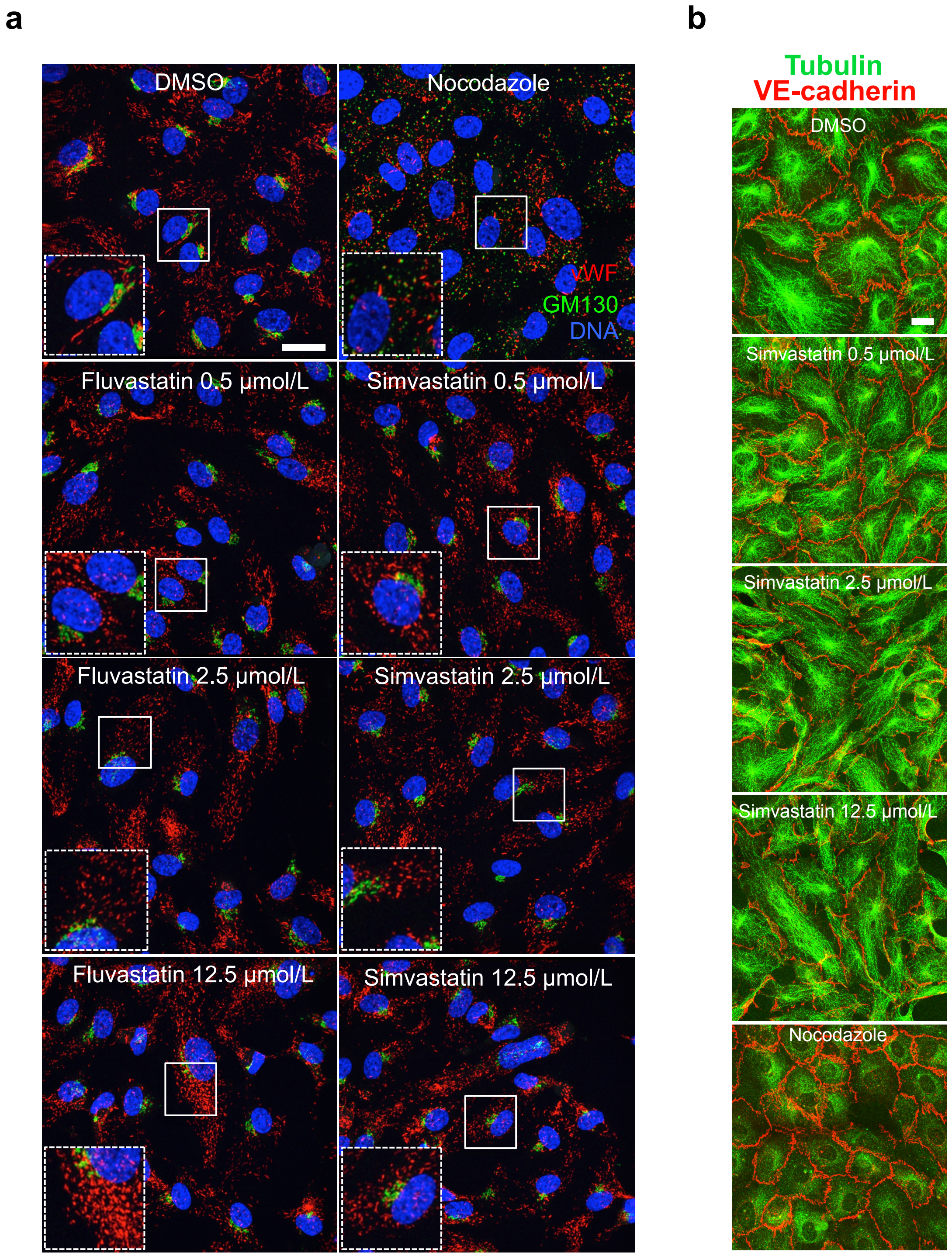

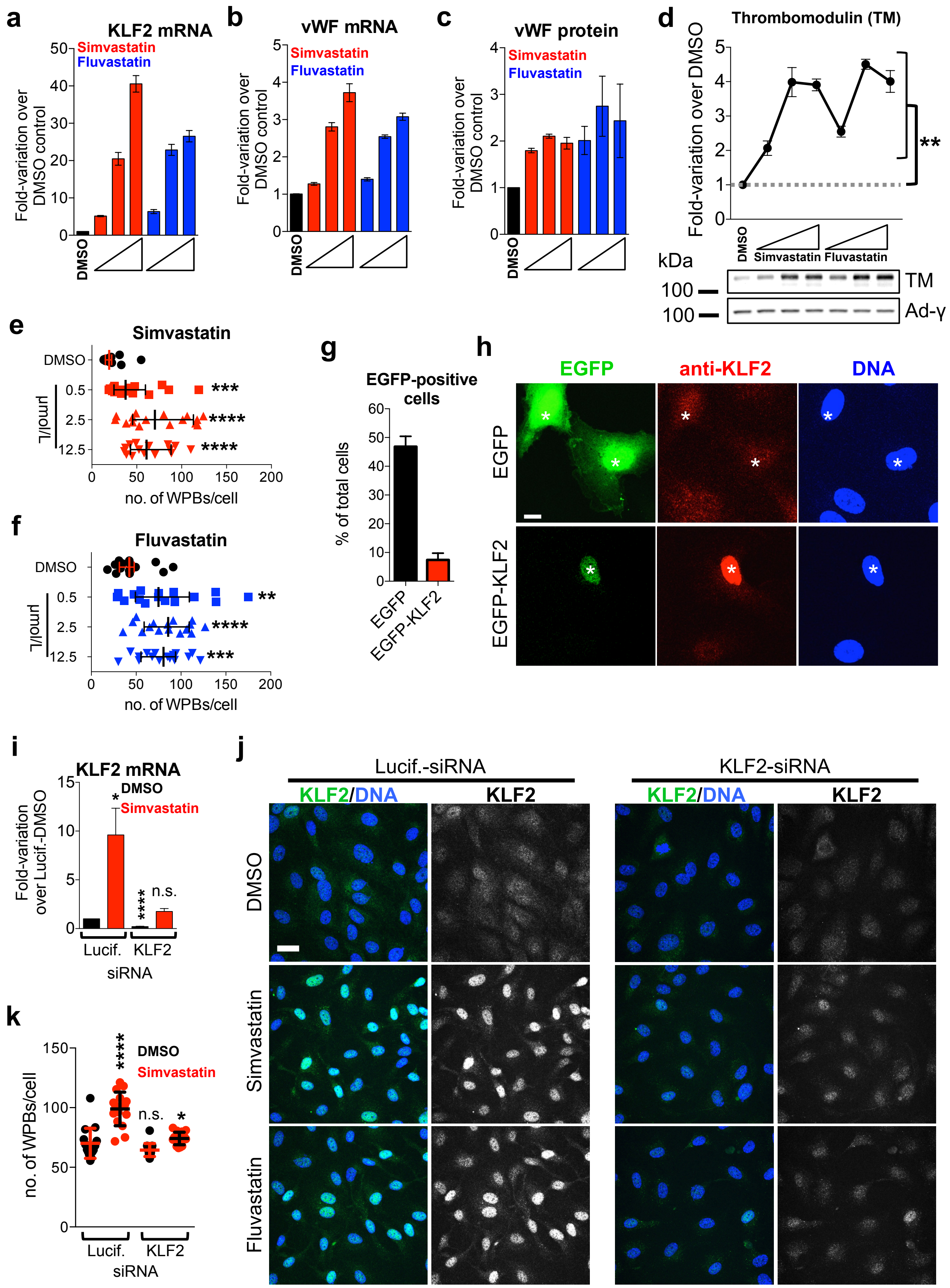
a
KLF2 mRNA
Simvastatin
Fluvastatin
Fold-variation over DMSO control
DMSO
b
vWF mRNA
c
vWF protein
d
Thrombomodulin (TM)
Fold-variation over DMSO
**
Simvastatin
Fluvastatin
kDa
100
TM
Ad-γ
e
Simvastatin
µmol/L
no. of WPBs/cell
f
Fluvastatin
g
EGFP-positive cells
% of total cells
EGFP
EGFP-KLF2
h
anti-KLF2
DNA
i
KLF2 mRNA
Fold-variation over Lucif.-DMSO
n.s.
Lucif.
KLF2
siRNA
j
Lucif.-siRNA
KLF2-siRNA
KLF2/DNA
KLF2
DMSO
Simvastatin
Fluvastatin
k
no. of WPBs/cell

Supplementary Figure 3

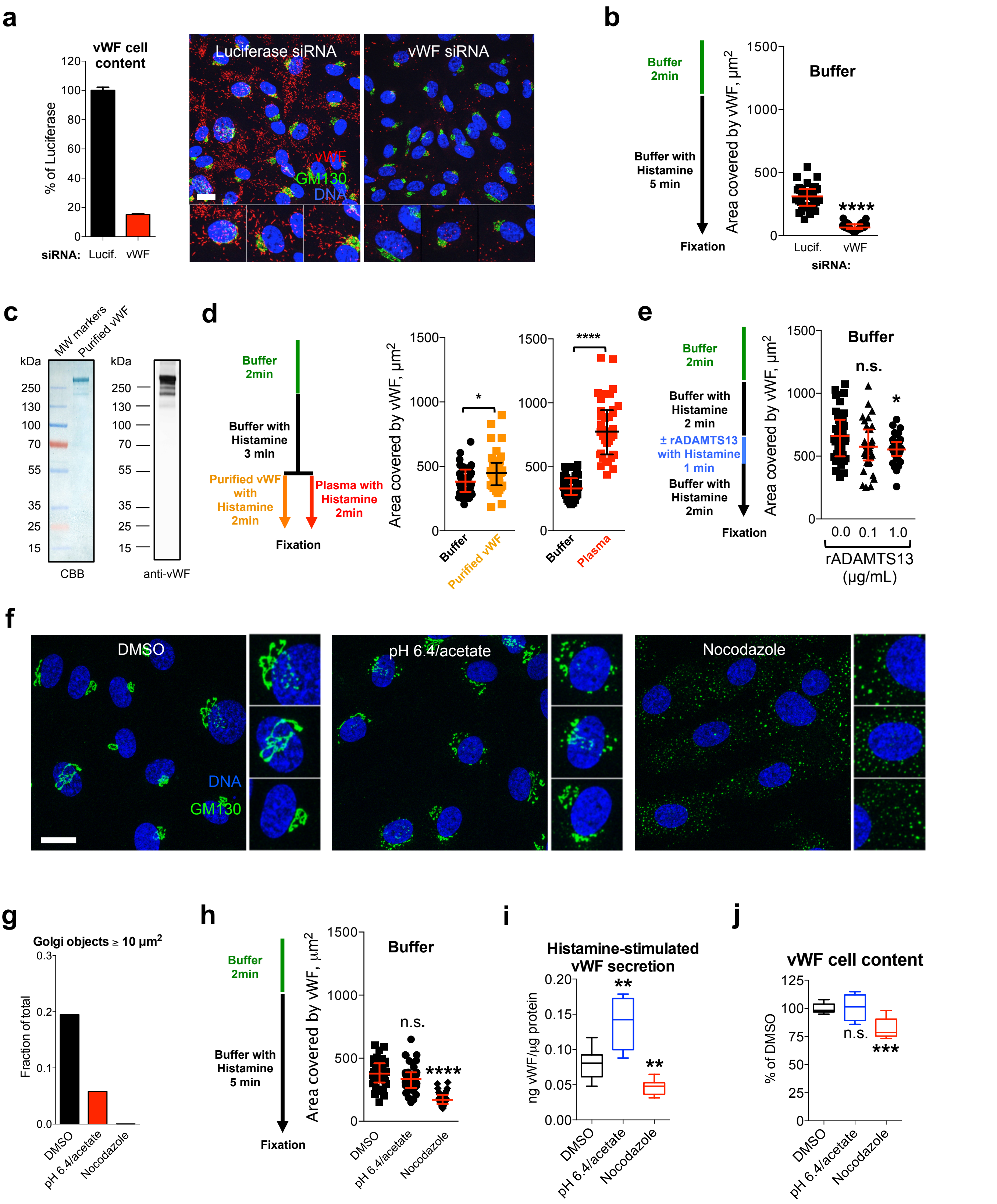

Supplement: Supplementary Information [file srep32473-s1.pdf]
